# Characterization of Nipah Virus from Naturally Infected *Pteropus vampyrus* Bats, Malaysia

**DOI:** 10.3201/eid1612.091790

**Published:** 2010-12

**Authors:** Sohayati A. Rahman, Sharifah S. Hassan, Kevin J. Olival, Maizan Mohamed, Li-Yen Chang, Latiffah Hassan, Norsharina M. Saad, Syamsiah A. Shohaimi, Zaini C. Mamat, M.S. Naim, Jonathan H. Epstein, Arshad S. Suri, Hume E. Field, Peter Daszak, the Henipavirus Ecology Research Group

**Affiliations:** Author affiliations: Veterinary Research Institute, Ipoh, Malaysia (S.A. Rahman, S.A. Shohaimi, Z.C. Mamat, M.S. Naim); Monash University, Selangor, Malaysia (S.S. Hassan);; EcoHealth Alliance, New York, New York, USA (K.J. Olival, J.H. Epstein, P. Daszak);; University of Science Malaysia, Kelantan, Malaysia (M. Mohamed);; University of Malaya, Kuala Lumpur, Malaysia (L.-Y. Chang);; University Putra Malaysia, Serdang, Selangor (L. Hassan, A.S. Suri); Ministry of Science, Technology and Innovation, Putrajaya, Malaysia (N.M. Saad);; Queensland Primary Industries and Fisheries, Moorooka, Queensland, Australia (H.E. Field)

**Keywords:** Nipah virus, viruses, isolation, characterization, Pteropus vampyrus, fruit bat, dispatch

## Abstract

We isolated and characterized Nipah virus (NiV) from *Pteropus vampyrus* bats, the putative reservoir for the 1998 outbreak in Malaysia, and provide evidence of viral recrudescence. This isolate is monophyletic with previous NiVs in combined analysis, and the nucleocapsid gene phylogeny suggests that similar strains of NiV are co-circulating in sympatric reservoir species.

Nipah virus (NiV) first emerged in Malaysia in 1998 (1), with subsequent human cases reported in Bangladesh (2) and India (3). Serologic and virologic evidence support the hypothesis that *Pteropus* spp. bats are the reservoir hosts for henipaviruses (4,5). However, the mechanisms of transmission between individual bats and of viral maintenance in a colony are poorly understood. We report isolation of NiV from *Pteropus vampyrus* bats, the putative reservoir for the 1998 outbreak in humans and pigs, and present evidence that these bats can harbor latent infections that recrudesce. Finally, we characterized this isolate and compared its phylogenetic position with all other known henipavirus sequences.

## The Study

We conducted a prospective cohort study from June 2004 through June 2005 on a group of 17 *P. vampyrus* flying foxes captured in 2 locations, using a nonrandom sampling method. Fourteen bats (73%) were from Lenggong (5°07′01.1′′N, 100°58′32.7′′E), and 3 bats (27%) were from Kampung Gajah (4°10′35′′N, 100°55′37′′E), Malaysia. This project was approved by the Wildlife Trust Institutional Animal Care and Use Committee, New York, New York, USA, and Department of Wildlife and National Park Malaysia research committee.

Because bats were included in the study in a staggered manner, each bat was monitored for antibody titer against NiV and virus excretion for 5 to 12 months. Bats were quarantined at Taiping Zoo (4°54′N, 100°45′E), Taiping, Malaysia, in a wire net (1 inch square) enclosure, 5 m long × 4 m wide × 3 m high; with a roof and cement floor. Inactivated bat serum specimens were screened for NiV antibodies by using a serum neutralization test. A titer of >8 was considered positive for specific antibodies against NiV, because bat serum is frequently toxic to Vero cells at higher concentrations (i.e., 1:2 or 1:4). A 4-fold increase in antibody titer was interpreted as an indication of an acute or recent infection (6). Seroreaction of juvenile bats was considered to represent NiV maternal antibody remnants.

During the study, 544 samples were cultured for virus isolation (272 throat and 272 urine or urogenital swab specimens). Samples were added to Vero cells (CRL 81; American Type Culture Collection, Manasssas VA, USA) and observed for characteristic syncytial type of cytopathic effects (1). NiV was isolated from only 1 sample, the urine of an adult female bat (no. 24). The antibody profile of this bat showed an antibody titer of 8 when tested on entry to the study, which later waned to negative (titer <4) on the second sampling. The bat remained antibody negative for 11 months, after which the bat again become seropositive; the titer rose from <4 to 32 over a 3-week period.

Virus isolation corresponded to the time when the antibody titer of the bat was on the verge of rising (<4 to 4). Two weeks later, 2 seronegative male bats (nos. 38 and 48) converted to a titer of 32; however, no virus was isolated. Details from our longitudinal serologic testing of these 3 bats are shown in Table 1. The isolation from bat no. 24 was confirmed as NiV as described (7). Serum neutralization test and virus isolation were performed in a BioSafety Level 3 Laboratory at Veterinary Research Institute, Ipoh, Malaysia.

**Table 1:** Longitudinal serologic test results from 3 *Pteropus vampyrus* bats that seroconverted while in captivity, June 2004–June 2005, Malaysia*

Date of test	Bat no. 24 (type AF)	Bat no. 38 (type JM)	Bat no. 48 (type JM)
2004 Jun 12	NE	16	NE
2004 Jul 8	8	<4	NE
2004 Jul 28	<4	<4	NE
2004 Aug 30	<4	<4	NE
2004 Oct 20	<4	<4	NE
2005 Jan 5	<4	<4	NE
2005 Mar 1	<4	<4	<4
2005 Mar 29	<4	<4	<4
2005 Apr 4	<4	<4	<4
2005 Apr 12	<4	<4	<4
2005 May 18	<4	<4	<4
2005 May 26	<4	<4	<4
2005 May 3	<4	<4	<4
2005 May 10	<4	<4	<4
2005 May 17	<4	<4	<4
2005 May 24	4	<4	<4
2005 May 31	16	<4	<4
2005 Jun 8	32	16	32

* Serum neutralization test titer of <4, negative; 4, inconclusive; 8–32, seroconverted. A, adult; F, female; J, juvenile; M, male; NE, animal not yet enrolled.

The sequence of NiV *P. vampyrus* (GenBank accession no. FN869553) and the alignment analysis show that NiV *P. vampyrus* differs from all known isolates from Malaysia at 98 nt positions; these nucleotide changes translated into amino acid changes at 44 positions. Subsequent analysis of the deduced amino acid sequences of the open reading frames of the nucleocapsid, phosphoprotein, matrix, fusion, attachment, and polymerase genes showed high sequence similarities (98%–99%) between nucleocapsid, matrix, fusion, attachment, and polymerase proteins of NiV *P. vampyrus* and other previously sequenced NiV isolates from Malyasia. However, phosphoprotein shares the lowest homology (96%). Table 2 shows a summary of the specific deduced amino acid changes compared with other NiV sequences.

**Table 2:** Summary of deduced amino acid changes in the N, P, M, F, G, and L proteins of NiV from *Pteropus vampyrus* bats compared with other NiV isolates*

Isolate†	N, aa position													P, aa position																															
NiV isolate	429		432					457						41				140				195					295					304				309					408			410	
*P. vampyrus*	**V**		**E**					**D**						**R**				**A**				**P**					**S**					**A**				**T**					**G**			**T**	
Human-CDC	I		G					N						Q				T				L					N					T				A					T			A	
*P. hypomelanus*	I		G					N						Q				T				L					N					T				A					T			A	
Pig-Tambun	I		G					N						Q				T				L					N					A				A					T			A	
	P, aa position																																												
NiV isolate	412			419					420				425				427				430					437					438				439					440				454	
*P. vampyrus*	**N**			**K**					**M**				**R**				**G**				**P**					**P**					**P**				**Q**					**S**				**P**	
Human-CDC	Y			N					V				S				A				H					Y					Q				E					G				T	
*P. hypomelanus*	Y			N					V				S				A				H					Y					Q				E					G				T	
Pig-Tambun	Y			N					V				S				A				H					Y					Q				E					G				T	
	P, aa position																								M, aa position														F, aa position						
NiV isolate	463	464					467				468					471				664					147				234					331					11				63		
*P. vampyrus*	**K**	**I**					**P**				**H**					**N**				**V**					**G**				**Y**					**V**					**S**				**A**		
Human-CDC	E	V					V				D					D				I					S				S					I					C				P		
*P. hypomelanus*	E	V					V				D					D				I					S				S					I					C				P		
Pig-Tambun	E	V					V				D					D				I					G				S					I					C				P		
	F, aa position					G, aa position																											L, aa position												
NiV isolate	460					20				186					426				444				470					481					223				1645					1753			2039
*P. vampyrus*	**K**					**N**				**D**					**I**				**V**				**Q**					**D**					**N**				**F**					**V**			**N**
Human-CDC	I					I				N					V				I				L					N					T				S					M			H
*P. hypomelanus*	I					I				N					V				I				L					N					T				F					M			H
Pig-Tambun	I					N				N					V				I				L					N					N				F					V			N

*N, nucleocapsid; P, phosphoprotein; M, matrix; F, fusion, G, attachment; L, polymerase; NiV, Nipah virus; CDC, Centers for Disease Control and Prevention. **Boldface** indicates amino acid changes. †GenBank accession nos.: NiV Human-CDC , AF212302; NiV *P. hypomelanus,* AF376747; NiV Pig-Tambun, AJ627196.

**Figure 1:**
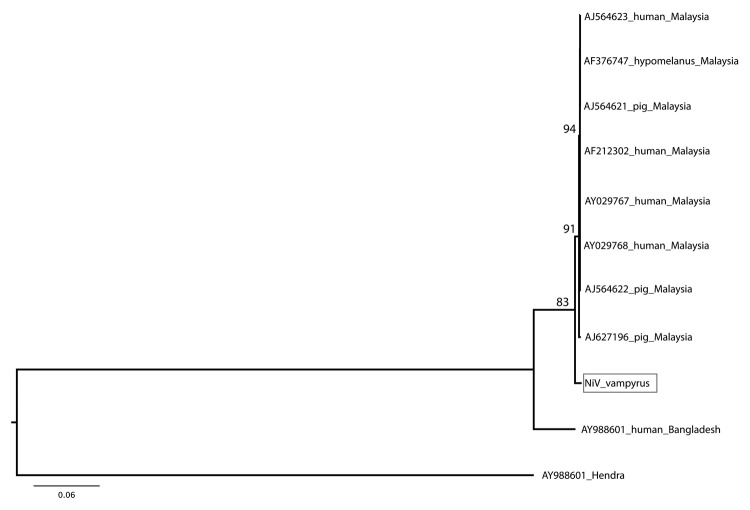
Phylogenetic position of Nipah virus (NiV) isolate from *Pteropus vampyrus* bats (box) in combined analysis of nucleocapsid, phosphoprotein, matrix, fusion, and attachment gene open reading frames (8.3 kb). Maximum likelihood tree, general time reversible + Γ model, 1,000 bootstrap replicates. NiV *P. vampyrus* is distinct but forms a clade with other NiV sequences from Malaysia, and the isolate from Bangladesh is more distantly related and basal to this group. GenBank accession numbers are shown for all comparison isolates; the polymerase gene is missing for AF376747 and thus that isolate is excluded from analysis. Scale bar indicates nucleotide substitutions per site.

**Figure 2:**
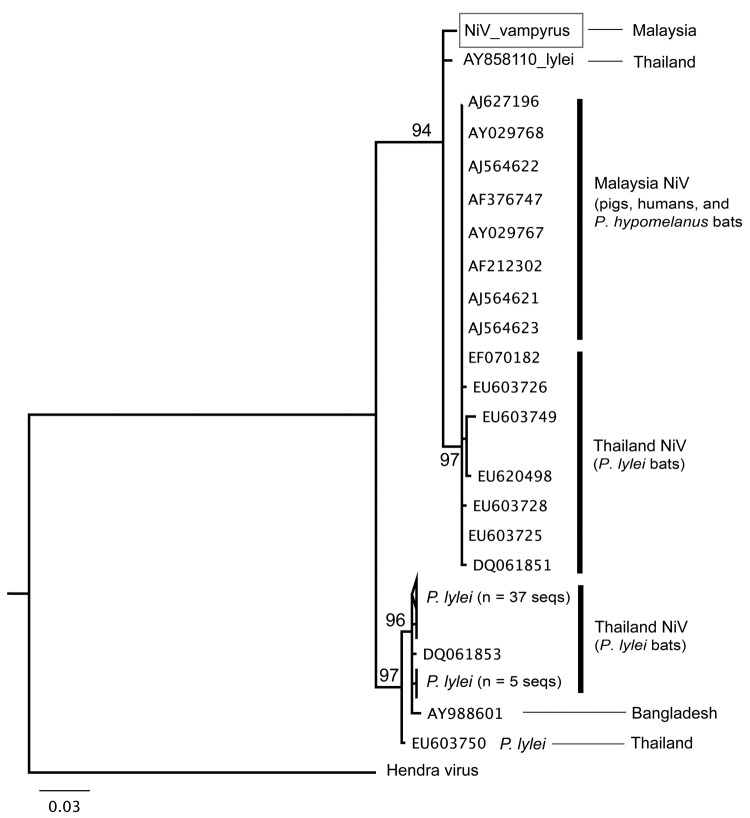
Partial nucleocapsid gene (358 bp) maximum-likelihood tree for all available Nipah virus (NiV) sequences (seqs) in GenBank, showing a high level of NiV sequence diversity in *Pteropus lylei* bat isolates from Thailand. NiV *P. vampyrus* (box) is most closely related to AY858110 from *P. lylei* bats and forms a large clade that includes other *P. lylei* bat isolates and all NiV sequences from Malaysia. GenBank accession numbers are given for NiV isolates from pigs in Malaysia (AJ627196, Tambun; AJ564621, Sg. Buloh; and AJ564622, Seremban), humans in Malaysia (AJ564623, AF212302, AY029767, and AY029768), humans in Bangladesh (AY988601), *P. hypomelanus* bats in Malaysia (AF376747), *P. lylei* bats in Cambodia (nucleocapsid gene-AY858110, DQ061851–58, EF070182–90, EU603724–58, EU620498, and EU624735–37), and Hendra virus from Australia (AF017149). Scale bar indicates nucleotide substitutions per site.

Phylogenetic analyses were generated by using maximum-likelihood methods (8). Sequences were analyzed with henipavirus sequences available in GenBank. The analysis of the combined nucleotide dataset shows that NiV *P. vampyrus* forms a monophyletic clade with other NiV isolates from Malaysia, yet it differs from human, pig, and *P. hypomelanus* bat isolates. NiV from humans in Bangladesh is more distantly related and basal to all NiV sequences from Malaysia (Figure 1). This relationship is further supported by the polymerase gene analysis (data not shown). When the nucleocapsid gene alone was analyzed, including 56 NiV sequences from *P. lylei* bats in Thailand, NiV *P. vampyrus* phylogenetically grouped most closely with NiV *P. lylei* (AY858110), and the monophyly of NiV sequences from Malysia was lost (Figure 2). This sister relationship between NiV *P. lylei* and NiV *P. vampryus* is also evident in analysis of the atttachment gene (data not shown).

## Conclusions

Our evidence suggests that NiV can recrudesce in previously infected adult bats, thus providing a new potential mechanism for maintenance in natural hosts. We isolated NiV from a seropositive adult bat at the time of capture, and therefore it was unlikely to have had remnant maternal antibodies. Also, it is unlikely that infection could have been introduced by other wild bats because other bats could not access the enclosure where the study bats were kept. The antibodies waned during the bat’s captivity, and subsequent seroconversion correlated with our finding of NiV in the individual bat’s urine. The bats were isolated from contact with wild bats, and all other bats placed in the colony were negative for henipavirus by culture; only bats 24, 38, and 48 subsequently seroconverted. Recrudescence of NiV infection in bats is not completely unexpected because NiV infection has resulted in (fatal) relapsing illness in humans, several months to 4 years after initial exposure (9). Other paramyxoviruses, including canine distemper (10) and measles virus (11), can persist in tissues for some years.

The seroconversion of the 3 bats in this colony is consistent with recent viral challenge (6) and a scenario in which bat 24 underwent recrudescence of a latent infection. The seroconversion supports the conclusion that bats 38 and 48 were infected through exposure to urine, feces, or saliva from bat 24.

NiV was not isolated from the 2 male bats, which may have been because of a low amount of virus excreted, a very narrow time frame for excretion, or both; however, these findings suggest that they did not undergo recrudescence. Evidence for NiV recrudescence adds to our understanding of henipavirus ecology and transmission dynamics. Repeated shedding of NiV through recrudescence may enhance viral maintenance in isolated colonies without the boom and bust dynamics, typical of acute viral infections with long-term immunity and reduce the necessity of intercolony migration for maintenance.

Our phylogenetic analyses help address some long-standing questions regarding the natural history of henipaviruses (12). Close homology between NiV *P. vampyrus* and a NiV *P. lylei* isolates and evidence from nucleocapsid and polymerase gene analysis suggest that NiV is naturally transmitted between these 2 species, which roost together in Thailand and parts of Cambodia. Furthermore, NiV diversity in isolates obtained from *P. lylei* bats demonstrates that multiple strains co-circulate within populations and that the ecology and sympatry of *Pteropus* spp., not co-evolutionary patterns, determine NiV strain diversity in reservoir hosts.

During the 1998 outbreak, NiV isolates from *P. hypomelanus* bats was found to be nearly identical to those from pigs and humans (5­6 nt changes) (13,14). However, this species is only found on offshore islands, has limited dispersal, and does not overlap with the index farms. Thus, *P. vampyrus* bats are likely the putative spillover hosts in Malaysia, not *P. hypomelanus* bats; nonetheless, our isolate differed markedly from others in the outbreak. Because laboratory contamination of the NiV *P. hypomelanus* isolate seems unlikely (14), the co-circulation of multiple strains in *P. vampyrus* bats is probable. Alternatively, the differences we observed in NiV *P. vampyrus* may be the result of rapid RNA virus evolution during the 7-year period between sampling of NiV *P. vampyrus* (2005) and sampling of the other isolates from Malaysia (1998–1999). Our data support this hypothesis. Assuming a known henipavirus genome length of ≈18,000 nt (15), the average substitution rate for *Paramyxoviridae* of 0.50 × 10^–3^ substitutions/site/year, and a constant molecular clock, the 98-nt changes observed correspond broadly to the time frame (≈7 years) between sampling of these isolates.

## References

[1] Chua KB, Bellini WJ, Rota PA, Harcourt BH, Tamin A, Lam SK, Nipah virus: a recently emergent deadly paramyxovirus. Science. 2000;288:1432–5. 10.1126/science.288.5470.1432 10827955

[2] Hsu VP, Hossain MJ, Paeashar UD, Ali MM, Ksiazek TG, Kuzmin I, Nipah virus encephalitis reemergence, Bangladesh. Emerg Infect Dis. 2004;12:2082–7. 15663842 10.3201/eid1012.040701 PMC3323384

[3] Chadha MS, Comer JA, Lowe L, Rota PA, Rollin P, Bellini WJ, Nipah virus–associated encephalitis outbreak, Siliguri, India. Emerg Infect Dis. 2006;12:235–40. 16494748 10.3201/eid1202.051247 PMC3373078

[4] Johara MY, Field H, Rashidi AM, Morrisi C, Vanderheide B, Rota P, Nipah virus infection in bats (order Chiroptera) in peninsular Malaysia. Emerg Infect Dis. 2001;7:439–41. 11384522 10.3201/eid0703.010312 PMC2631791

[5] Olson J, Rupprecht C, Rollin P, An U, Niezgoda M, Clemins T, Antibody to Nipah-like virus in bats (*Pteropus lylei*), Cambodia. Emerg Infect Dis. 2002;8:987–8. 12194780 10.3201/eid0809.010515 PMC2732552

[6] Thrusfield M. Veterinary epidemiology, 3rd ed. Oxford (UK): Blackwell Science Ltd; 2005.

[7] Maizan M, Mohd Ali AR, Sharifah SH. The identification and distinction between Nipah virus and Hendra virus by using RT-PCR, sequencing and restriction enzyme analysis. Asia Pac J Mol Biol Biotechnol. 2000;8:101–6.

[8] Stamatakis A, Hoover P, Rougemont J. A rapid bootstrap algorithm for the RAxML web-servers. Syst Biol. 2008;75:758–71. 10.1080/10635150802429642 18853362

[9] Chong HT, Tan CT. Relapse and late-onset Nipah virus encephalitis: a report of three cases. Neurological Journal of Southeast Asia. 2003;8:109–12.

[10] Tan CT, Goh KJ, Wong KT, Sarji SA, Chua KB, Chew NK, Relapsed and late-onset Nipah encephalitis. Ann Neurol. 2002;51:703–8. 10.1002/ana.10212 12112075

[11] Payne FE, Baublis JV, Itabashi HH. Isolation of measles virus from cell cultures of brain from a patient with subacute sclerosing panencephalitis. N Engl J Med. 1969;281:585–9. 10.1056/NEJM196909112811103 4980073

[12] Field H, Young P, Yob JM, Mills J, Hall L, Mackenzie J. The natural history of Hendra and Nipah viruses. Microbes Infect. 2001;3:307–14. 10.1016/S1286-4579(01)01384-3 11334748

[13] AbuBakar S, Chang LY, Ali AR, Sharifah SH, Yusoff K, Zamrod Z. Isolation and molecular identification of Nipah virus from pigs. Emerg Infect Dis. 2004;10:2228–30. 15663869 10.3201/eid1012.040452 PMC3323361

[14] Chua KB, Koh CL, Hooi PS, Wee KF, Khong JH, Chua BH, Isolation of Nipah virus from Malaysian Island flying-foxes. Microbes Infect. 2002;4:145–51. 10.1016/S1286-4579(01)01522-2 11880045

[15] Jenkins GM, Rambaut A, Pybus OG, Holmes EC. Rates of molecular evolution in RNA viruses: a quantitative phylogenetic analysis. J Mol Evol. 2002;54:156–65. 10.1007/s00239-001-0064-3 11821909

